# A Lightweight Framework For Chromatin Loop Detection at the Single‐Cell Level

**DOI:** 10.1002/advs.202303502

**Published:** 2023-10-10

**Authors:** Fuzhou Wang, Hamid Alinejad‐Rokny, Jiecong Lin, Tingxiao Gao, Xingjian Chen, Zetian Zheng, Lingkuan Meng, Xiangtao Li, Ka‐Chun Wong

**Affiliations:** ^1^ Department of Computer Science City University of Hong Kong Kowloon Tong Hong Kong SAR; ^2^ BioMedical Machine Learning Lab, Graduate School of Biomedical Engineering University of New South Wales Sydney 2052 Australia; ^3^ Molecular Pathology Unit, Center for Cancer Research, Massachusetts General Hospital Department of Pathology Harvard Medical School Boston MA 02129 USA; ^4^ Department of Computer Science The University of Hong Kong Pok Fu Lam Hong Kong SAR; ^5^ Department of Medical Biophysics, Faculty of Medicine University of Toronto Toronto Ontario M5G1L7 Canada; ^6^ School of Artificial Intelligence Jilin University Changchun 130012 China

**Keywords:** chromatin loops, functional loops, multi‐connected hubs, single‐cell Hi‐C

## Abstract

Single‐cell Hi‐C (scHi‐C) has made it possible to analyze chromatin organization at the single‐cell level. However, scHi‐C experiments generate inherently sparse data, which poses a challenge for loop calling methods. The existing approach performs significance tests across the imputed dense contact maps, leading to substantial computational overhead and loss of information at the single‐cell level. To overcome this limitation, a lightweight framework called scGSLoop is proposed, which sets a new paradigm for scHi‐C loop calling by adapting the training and inferencing strategies of graph‐based deep learning to leverage the sequence features and 1D positional information of genomic loci. With this framework, sparsity is no longer a challenge, but rather an advantage that the model leverages to achieve unprecedented computational efficiency. Compared to existing methods, scGSLoop makes more accurate predictions and is able to identify more loops that have the potential to play regulatory roles in genome functioning. Moreover, scGSLoop preserves single‐cell information by identifying a distinct group of loops for each individual cell, which not only enables an understanding of the variability of chromatin looping states between cells, but also allows scGSLoop to be extended for the investigation of multi‐connected hubs and their underlying mechanisms.

## Graphical abstract

A lightweight framework called scGSLoop is introduced to detect chromatin loops on single‐cell Hi‐C data. Leveraging graph‐based deep learning, scGSLoop operates on sparse matrices, avoiding resource‐intensive data densification. This model not only achieves computational efficiency, but also accurately predicts loops genome‐wide. It preserves single‐cell information, enabling the investigation of looping variability and multi‐connected hubs.

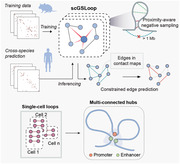

## 1 Introduction

Over the past two decades, the emergence of genome‐wide DNA positioning techniques has significantly improved our understanding of the principles underlying chromatin folding. Sequencing‐based technologies such as Hi‐C^[^ ^1^, ^2^ ^]^ have revolutionized our ability to capture the average genome patterns in 3D space across a large cell population. The advancement of these technologies has led to the establishment of a multi‐layered perspective on the organization of the 3D genome,^[^ ^3^, ^4^, ^5^ ^]^ that the hierarchy of the 3D structure is composed of multiple levels of architectural units, including chromatin loops,^[^ ^2^ ^]^ topologically associating domains (TADs),^[^ ^6^, ^7^ ^]^ and compartments.^[^ ^1^, ^2^ ^]^

In spite of the significant strides made by Hi‐C and other C‐like technologies in uncovering the mysteries of the chromatin interactome, the issue of cell‐to‐cell variability in this regard remains an area of fascination for researchers. This has led to the development of new technologies with single‐cell resolution. Single‐cell Hi‐C (scHi‐C)^[^ ^8^, ^9^, ^10^, ^11^, ^12^, ^13^, ^14^ ^]^ has been devised to capture the heterogeneity of the 3D genome between cells. It has been confirmed by scHi‐C that chromatin structures in individual cells can differ from the average structures detected by bulk Hi‐C and can vary across cells.^[^ ^15^, ^16^ ^]^ Moreover, it has also been revealed that the cells undergoing dynamic biological processes such as development,^[^ ^17^ ^]^ differentiation,^[^ ^18^ ^]^ reprogramming,^[^ ^12^ ^]^ and cell cycle progression^[^ ^9^ ^]^ usually exhibit significant changes in their chromatin organization.

Although scHi‐C has the potential to deepen our understanding of the 3D organization of the eukaryotic genome, the technology is not without its challenges, as data generated in scHi‐C experiments typically suffer from extreme sparsity.^[^ ^19^, ^20^ ^]^ Despite continuous efforts to improve the experimental protocols, this sparsity remains a persistent obstacle that is unlikely to disappear. This is due to the limited number of restriction sites in a nucleus, which imposes a theoretical upper bound on interactions that can be captured, as discussed in ref. [19]. Therefore, the computational methods for analyzing such data must be capable of handling its sparseness. One common practice adopted in previous studies for calling structural features^[^ ^20^, ^21^, ^22^, ^23^ ^]^ is to densify the contact maps using imputation techniques, and then detect architectural units on the densified matrix in a straightforward manner. This approach requires feature calling methods to be applied to dense matrices, which can be computationally intensive. In particular, existing loop calling methods^[^ ^21^, ^22^ ^]^ further involve performing significance tests across the imputed matrices of all cells to identify loop candidates. The requirement for both dense imputation and significance test together leads to a highly resource‐demanding process.^[^ ^24^ ^]^ Moreover, the significance test‐based loop calling results in the definition of loops being dependent on cell types, where only a shared set of loops can be generated for each cell type, causing information at the single‐cell level to be lost.

Here, we introduce scGSLoop (using graph information and sequence features to detect loops on single‐cell Hi‐C), a lightweight framework that can efficiently and accurately identify chromatin loops for each cell in scHi‐C datasets. ScGSLoop was designed to directly operate on the extremely sparse scHi‐C data and can predict chromatin loops for each cell in a large dataset within a short amount of time. This framework not only addresses the challenges posed by the massive volume of data but also provides more accurate loop detection than the current state‐of‐the‐art computational tool for scHi‐C data.^[^ ^21^ ^]^ Furthermore, cross‐species validations demonstrate that the proposed model is highly generalizable and able to predict chromatin interactions in unseen scHi‐C datasets regardless of experimental platform. As a loop caller that can detect both structural and functional loops over the whole genome, scGSLoop may serve as a valuable tool for researchers seeking to gain insights into chromatin organization and its implications for genome behavior and functions. Unlike existing tools for scHi‐C loop calling, scGSLoop can decipher chromatin looping states at the single‐cell level, providing more direct information about the variability of chromatin organization across individual cells. The unique features of the single‐cell loops detected by scGSLoop have facilitated the discovery of a series of multi‐connected hubs, which further support the idea of the critical involvement of the transcription factor KLF4 in the mediation of hubs in the 3D genome.

## 2 Results

### 2.1 Efficient scHi‐C Loop Calling Using Lightweight Machine Learning

The interaction matrices generated by scHi‐C exhibit a high degree of sparsity and can be effectively represented as a graph structure consisting of linearly connected nodes. This linear connectivity of genomic bins injects sequence information into the data, providing a perspective from the 1D sequence standpoint. To both take advantage of the sparsity of contact maps and utilize sequence information in scHi‐C data, we developed a novel variational graph autoencoder (VGAE) called the proximity‐aware constrained VGAE (PC‐VGAE). This model integrates two specialized mechanisms for scHi‐C data to improve the learning and inferencing rules of the model (Section 3). The first mechanism is proximity‐aware negative sampling, which limits the negative sampling process to potential edges that are within reasonable 1D distances on the genome, allowing for better capture of the local structure of the genomic context. The second mechanism is constrained edge prediction, which restricts the predicted edges to the existing contacts on the interaction matrices. This mechanism narrows down the searching space to a small subset of potential loop candidates, enabling efficient and effective sparse inferencing. With PC‐VGAE, scGSLoop is able to directly operate on the extremely sparse scHi‐C data and accurately predict loops on each individual cell.

In addition to the algorithmic design of the model, scGSLoop further takes in the sequence information by learning from the positional features and static DNA features of genomic loci. The model utilizes statistics of k‐mer and CTCF motifs extracted from genome assemblies as node features, providing essential knowledge for graph‐based learning. On top of that, we utilized a simple yet effective positional encoding scheme^[^ ^25^ ^]^ to inject genomic coordinate information to the model.

While PC‐VGAE is capable of handling sparse graph structures, when dealing with scHi‐C data that is too low in density, the resulting limited number of loop candidates may adversely affect the model's performance. To address this issue, we have developed an optional augmentation module that slightly increases the density of the contact maps. This augmentation process helps to generate contact maps with a greater number of loop candidates, while still maintaining their sparsity (Section 3).

**Figure 1:**
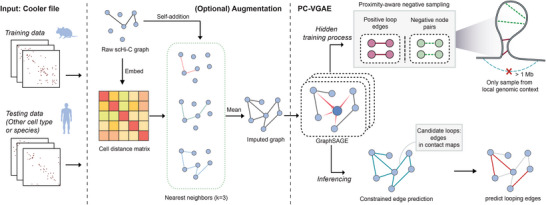
Architecture of scGSLoop framework. The framework accepts .scool files (a format associated with Cooler) as input and consists of two computational stages. The optional augmentation module can enhance data that is of insufficient quality, thereby increasing the number of loop candidates. The PC‐VGAE module is responsible for loop calling in this framework, and it leverages local genomic contexts through proximity‐aware negative sampling. The module predicts loop edges using a constrained edge prediction mechanism.

In this study, each scGSLoop model was trained on one dataset and tested on another independent scHi‐C dataset, and our experiments demonstrated that scGSLoop outperformed the state‐of‐the‐art scHi‐C loop caller SnapHiC^[^ ^21^ ^]^ in terms of both accuracy and computational efficiency. **Figure** **1** provides the reader with a comprehensive overview of the architecture of scGSLoop.

### 2.2 scGSLoop Recovers Loops from Bulk Hi‐C Experiments

**Figure 2:**
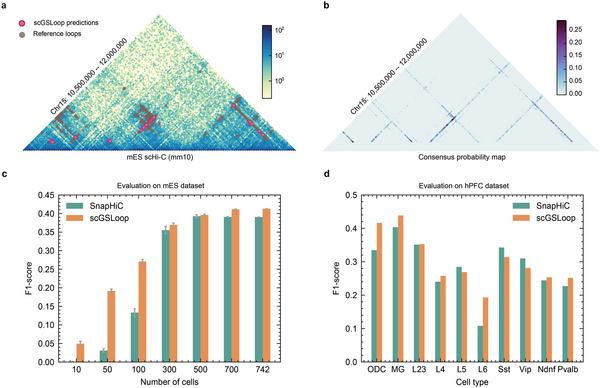
ScGSLoop detects chromatin loops at high accuracy. a) The consensus loops predicted by scGSLoop demonstrate consistency with the loops detected on bulk Hi‐C. b) Probability map generated by scGSLoop. c) F1 scores of scGSLoop and SnapHiC, evaluated on different numbers of mES cells. d) F1 scores of scGSLoop and SnapHiC, evaluated on different cell types of the hPFC dataset. ODC: oligodendrocyte progenitor cells; MG: microglia; L2/3, L4, L5, L6, Sst, Vip, Ndnf, and Pvalb are neuron subtypes.

We sought to illustrate the validity of the chromatin loops detected by scGSLoop by aligning these putative loops with the loops detected on bulk Hi‐C contact maps. To ensure fairness and effectiveness of the validation results, we adopted a cross‐species configuration that we trained an scGSLoop model on the single‐nucleus methyl‐3C‐seq (sn‐m3C‐seq) dataset generated from human prefrontal cortex (hPFC)^[^ ^14^ ^]^ and used that model to predict loops on an independent scHi‐C dataset obtained from mouse embryonic stem (mES) cells.^[^ ^9^ ^]^ By aggregating probability maps of multiple individual cells predicted by scGSLoop, we obtained a consensus set of loops on mES scHi‐C to examine the performance of scGSLoop in terms of recovering high‐confidence loops detected on bulk experiments, and further benchmark it against SnapHiC. We found that the pattern of chromatin loops detected by scGSLoop is highly consistent with the loop calls derived from bulk Hi‐C (**Figure** **2**a). The consensus probability map was also visualized to characterize the ability of scGSLoop to measure the importance of the contacts in the dataset (Figure 2b). The fine‐grained probability map generated by scGSLoop highlights the advantage of scGSLoop over existing statistical models that it can output a continuous score for each potential loop, potentially providing more insights into the likelihood and strength of interactions between genomic regions.

To investigate the performance of scGSLoop under different dataset conditions, we sub‐sampled the 742‐cell mES dataset to six subsets of different numbers of cells (10, 50, 100, 300, 500, and 700). Our experiments showed that scGSLoop consistently achieved higher F1 scores than SnapHiC across all sub‐sampled datasets as well as the complete dataset (Figure 2c). Furthermore, scGSLoop performed particularly well when the number of cells was low, whereas SnapHiC recognized fewer loops (Tables S1 and S2, Supporting Information) and experienced severe performance decay due to the small number of cells available for carrying out significance tests.

We performed an additional evaluation via training the model on the mES dataset and predicting on the hPFC dataset, which is composed of multiple cell types, to further validate the ability of scGSLoop to annotate loops on various human cell types. Our evaluation, shown in Figure 2d, demonstrated that scGSLoop outperformed SnapHiC with higher F1 scores achieved in seven out of ten cell types evaluated in hPFC cells.

The experimental conditions and species of origin for the training sets and test sets used in these comparisons varied significantly, and there was large discrepancy between the number of contacts present in the two datasets, with the hPFC dataset having a much higher median contact count of 1 514 790 compared to the mES dataset's median contact count of 340 716.5. Despite these differences, scGSLoop demonstrated superior performance, indicating its remarkable generalizability. This suggests that scGSLoop has the potential to be effectively applied to diverse scHi‐C datasets generated under different conditions and with different sequencing depths, enabling accurate detection of chromatin interactions. In addition, the model's performance remained largely preserved when tested on a further downsampled mES dataset (Figure S1, Supporting Information), which only contained 10% as many contacts as the training set (i.e., the hPFC dataset). These experimental findings provide additional evidence of the model's robustness against different sequencing depth, further highlighting its usability for various data qualities.

### 2.3 Time and Memory Efficiency of scGSLoop

**Figure 3:**
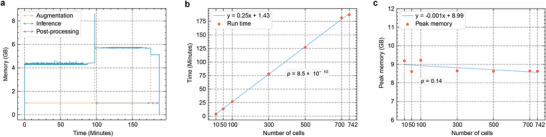
Memory and time consumption of scGSLoop. a) Memory profile of scGSLoop evaluated on the 742‐cell mES dataset. b) A plot of the time consumption of scGSLoop as a function of the number of cells. The resulting linear regression line is represented by the blue line. *p*‐value is calculated using a two‐sided Wald Test. c) A plot of the peak memory consumption of scGSLoop as a function of the number of cells. *p*‐value is calculated using a two‐sided Wald Test. Regarding (b) and (c), we accept the alternative hypothesis that the slope of the regression line is nonzero if the two‐sided Wald Test yields a *p*‐value less than 0.05; otherwise, we accept the null hypothesis that the slope is zero.

Assessing the computational resources and running time required by a bioinformatics tool is essential for evaluating its usability. To evaluate the efficiency of scGSLoop, we first profiled its time and memory usage on the 742‐cell mES dataset to examine its computational efficiency. The results are shown in **Figure** **3**a for memory usage and time consumption of a single run of scGSLoop inference. The peak memory usage was less than 9 GB, which is within the capacity of most workstations. Additionally, the total running time, including augmentation, loop annotation, and I/O overhead, was less than 3.5 h, indicating that scGSLoop is a highly efficient tool for analyzing large‐scale single‐cell Hi‐C datasets.

To assess the scalability of scGSLoop with respect to dataset size, we then conducted further investigations. Figure 3b provides a visual representation of the time consumption of scGSLoop as a function of increasing dataset size. The plot shows that the time consumption grows linearly with the dataset size (two‐sided Wald Test *p* < 0.05). Moreover, to examine the memory usage of scGSLoop with varying dataset sizes, we plotted the memory consumption against the dataset cardinality, as shown in Figure 3c. Different from the time consumption, the memory usage remains relatively constant as the dataset size increases (*p*‐value > 0.05). This stability in memory consumption enables scGSLoop to scale efficiently with large‐scale scHi‐C datasets.

It is important to note that, in the memory and time profiling experiments mentioned above, we restricted the number of PyTorch workers used for data fetching to one, ensuring the program operates strictly in single‐process mode. However, in practical usage, users can choose to increase the number of processes to reduce the overall run time. Moreover, if multiple GPUs are available, dividing the dataset into smaller subsets and running scGSLoop in parallel can further accelerate the inference process. This approach can be particularly effective when working with large datasets.

The computational efficiency of scGSLoop addresses the long‐standing problem in scHi‐C loop calling. The ability of scGSLoop to annotate loops directly on sparse maps, made possible by the design of PC‐VGAE, is a significant advancement in single‐cell loop calling. This unique approach eliminates the need to densify interaction matrices, unlike existing pipelines such as SnapHiC, which can lead to heavy memory burden and/or time consumption. For example, in the experiments in SnapHiC,^[^ ^21^ ^]^ the authors used fifteen compute nodes each with three processors to run the algorithm in parallel. 96 GB memory was allocated to each node to prevent memory overflow. Despite such abundant computational resources, it still took more than 20 h to detect loops on a smaller dataset containing 400 cells. A later version of SnapHiC, namely SnapHiC2,^[^ ^22^ ^]^ improved the computational efficiency of the model by introducing a sliding window mechanism for the imputation procedure. Despite the sharply reduced resource demand, in our local experiment with six processes on a small dataset containing 100 mES cells, SnapHiC2 still consumed a large amount of processing time (14.8 h) and a relatively high amount of peak memory usage (25.5 GB). In contrast, scGSLoop is highly scalable and can detect loops efficiently, making it an ideal tool for scHi‐C loop calling.

These results demonstrate that scGSLoop is capable of processing large and complex datasets in a reasonable amount of time and with moderate hardware requirements, making it a valuable tool for researchers exploring the intricate architecture of chromatin in single cells.

### 2.4 scGSLoop Detects Both Structural and Functional Loops

**Figure 4:**
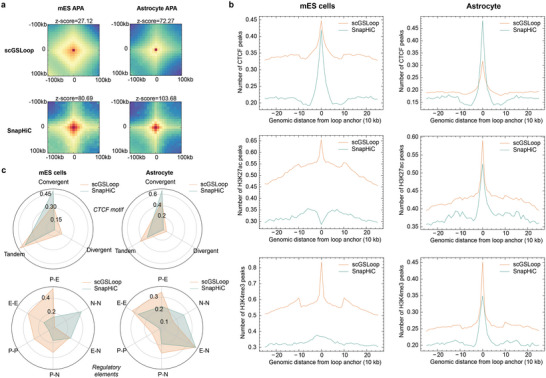
Structural and functional profiles of loops predicted by scGSLoop and SnapHiC. a) APA plots of loops predicted by both models in mES cells and astrocyte cells. b) Enrichment of CTCF, H3K27ac, and H3K4me3 ChIP‐Seq peaks surrounding the loop anchors predicted by both models in mES and astrocyte cells. A 250 kb genomic context was used upstream and downstream for profiling the ChIP‐Seq peaks. c) Upper part: orientations of CTCF motifs of the loops annotated by both models; lower part: *cis*‐regulatory elements at both anchors of loops. P, promoter; E, enhancer; N, None.

We sought to investigate the aggregate profiles of the scGSLoop consensus loops in mES and astrocyte and compared them with the loops predicted by SnapHiC. We performed an aggregate peak analysis (APA) (**Figure** **4**a) and observed that both SnapHiC and scGSLoop generated loops that exhibited considerable enrichment against the lower left corner (*z*‐score > 1.64). Despite the remarkable performance mentioned in the previous section, the loops generated by scGSLoop were found to exhibit lower significance levels compared to those generated by SnapHiC.

To further scrutinize this difference, we examined the structural and functional characteristics of the predicted loops, that CTCF, H3K27ac, and H3K4me3 peaks captured by ChIP‐Seq were collected and aggregated over ±250 kb of all putative loop anchors for both cell types (Figure 4b). Although the CTCF signals on the scGSLoop‐predicted loops in astrocyte cells were less enriched than those detected by SnapHiC, it is shown that loops annotated by scGSLoop were more frequently associated with transcription‐related active elements (i.e., H3K27ac and H3K4me3 are more enriched around loop anchors detected by scGSLoop). Interestingly, the loops predicted by SnapHiC on the mES dataset showed no discernible enrichment for H3K27ac or H3K4me3. Moreover, the aggregate profile of H3K27ac signals around SnapHiC‐predicted loops displayed an unusual pattern. Despite this, SnapHiC still succeeded to annotate loops that matched the expected patterns on astrocyte cells. The discrepancy in epigenetic profiles on anchors predicted by SnapHiC between mES and astrocyte cells suggests that there may be potential issues with SnapHiC's ability to detect epigenetically consistent loops for different experimental protocols. In contrast, scGSLoop is generalizable across different species and experimental protocols, delivering better predictive ability in detecting functional loops (promoter‐enhancer loops). We reason that SnapHiC may focus more on stable structural loops mediated by CTCF and cohesin, while neglecting the functional loops that may not be as visually prominent on the dense contact maps.^[^ ^26^ ^]^ The loops predicted by scGSLoop, however, are likely to contain a larger group of functional loops, which also explains their lower significance level of APA and the less pronounced enrichment of CTCF binding.

To provide more evidence on this hypothesis, we aligned the genomic positions of CTCF motifs and the ChromHMM^[^ ^27^ ^]^ segmentation of *cis*‐regulatory elements (i.e., promoters and enhancers) with the loops that were annotated using scGSLoop and SnapHiC. This allowed us to characterize the structural and functional elements on both sides of the loops. Figure 4c illustrates that the properties of the captured interactions were consistent with the discoveries made using 1D ChIP‐Seq data. Despite being associated with fewer convergent CTCF motifs, the loops detected by scGSLoop still exhibited the expected motif profile, where convergent and tandem loops were more prevalent than divergent ones (Figure 4c, upper part). It was also demonstrated that loops detected by scGSLoop were more likely to harbor *cis*‐regulatory elements on both sides (Figure 4c, lower part). In this experiment, scGSLoop identified a prevalence of promoter‐enhancer loops, whereas SnapHiC captured a much higher proportion of loops with at least one anchor lacking a regulatory element. Particularly, among the loops predicted by SnapHiC in mES cells, those with regulatory elements depleted on either side were even found to be the predominant type. Another piece of supporting evidence arises from the increasingly widespread consensus that, as a general rule, functional loops tend to exhibit shorter genomic distances between anchors compared to structural loops.^[^ ^5^, ^28^ ^]^ We had anticipated that a higher proportion of functional loops in the predictions would result in a skewed distribution of loop sizes toward shorter genomic distances. Accordingly, we analyzed the size distributions of the loops predicted by both models (Figure S2, Supporting Information), and our findings were in agreement with the expectations. Overall, these results, along with the earlier findings presented in this section, illustrate that scGSLoop is more effective in identifying functional loops, while also showing a degree of capability in identifying structural loops.

### 2.5 scGSLoop Captures Loop Consistency and Variability on Single‐Cell Level

Single‐cell Hi‐C is a technique that captures the 3D organization of individual cells and provides information on cell‐to‐cell variability. However, previous computational tools, such as the SnapHiC pipeline,^[^ ^21^ ^]^ have limitations in their ability to analyze single‐cell Hi‐C data. These tools can only generate a consensus set of loops for each cell type, ignoring cell‐to‐cell variability. ScGSLoop, on the other hand, is an advanced tool that can detect chromatin loops at the single‐cell level, enabling a more comprehensive understanding of genome structure. By analyzing single‐cell loops predicted by scGSLoop in mES cells, we were able to successfully recover both known architectural consistency and heterogeneity across different cells in the dataset.

**Figure 5:**
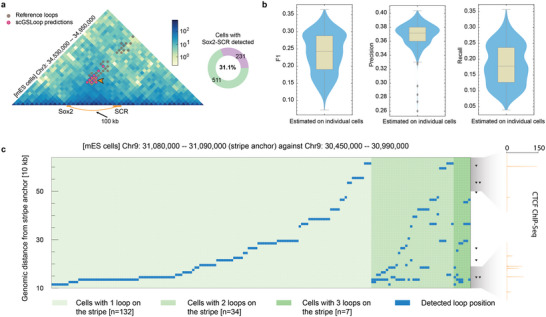
Evaluation of single‐cell loops predicted by scGSLoop. a) Highly robust *Sox2*‐SCR loop was detected in 31.1% cells. b) F1 score, precision, and recall of single‐cell loops. c) Single‐cell loops were detected in 173 cells in the stripe domain studied in ref. [29]. The blue points indicate the genomic distances from the stripe anchor where looping occurs. The shade of green reflects the number of loop occurrences in each cell. In the majority of cells, scGSLoop identifies a single loop associated with the stripe anchor. The black triangles on the right side represent the positions of CTCF motifs on the positive strand. The CTCF ChIP‐Seq peaks are denoted beside the CTCF motif track. Unmarked loci represent genomic regions where there are no ChIP‐Seq peaks.

We first examined the ability of scGSLoop in recovering a robust loop in mES cells that is essential for the expression of *Sox2* gene. This gene encodes the transcription factor SOX2, which plays a vital role in maintaining the pluripotency of mES cells. Furthermore, the *Sox2* gene is known to be regulated by a downstream enhancer cluster named *Sox2* control region (SCR), which has been shown to interact with the promoter in a loop configuration and contribute to 95% of SOX2 expression.^[^ ^30^, ^31^, ^32^ ^]^ This promoter‐enhancer loop has been demonstrated to be highly robust in recent research,^[^ ^33^ ^]^ as it is able to bypass CTCF boundaries to maintain the proper functioning of *Sox2*. **Figure** **5**a illustrates that the interactions between *Sox2* and SCR are identified and mapped to the consensus loop set of the mES dataset. At the single‐cell level, we observed that this robust *Sox2*‐SCR loop was present in 31.1% (231 out of 742) of individual cells (Figure 5a, right part). These results align with the expectation that stable regulatory loops with important biological functions should occur frequently across cells.

The loop extrusion model^[^ ^15^ ^]^ pictures the formation of chromatin loops as a highly dynamic process. In particular, architectural stripes are considered as a result of asymmetric extrusion mediated by cohesin and CTCF,^[^ ^29^ ^]^ where one subunit of cohesin slides along the domain (i.e., a stripe domain^[^ ^29^, ^34^ ^]^), and the other subunit is halted on a CTCF anchor. This mechanism leads to stripe patterns on bulk Hi‐C contact maps, with stripes representing the statistical ensemble of the dynamic interactions in different cells caused by the extrusion process. Architectural stripes provide a useful model to examine the ability of scGSLoop to capture cell‐to‐cell variability across the dataset. We selected an architectural stripe in mES cells studied in ref. [29] and inspected the loops detected in each cell associated with the stripe anchor. Figure 5c demonstrates the distance between the looping regions and the stripe anchor for each cell. Additionally, we also presented the number of loops detected in each cell in the same figure. We found that scGSLoop captured one loop in most cells, while two or three loops were detected in a smaller proportion of cells. The loops detected by scGSLoop were observed across all distances from the stripe anchor, consistent with both the aggregated observation on the bulk Hi‐C contact map and the asymmetric extrusion hypothesis for architectural stripe formation. In particular, our observations have shown that there is a region‐specific preference for chromatin looping, as a larger proportion of cells formed loops at loci located ≈130–150 kb away from the stripe anchor. By aligning the domain with CTCF motifs and ChIP‐Seq profiles (Figure 5c, right side), we demonstrated that the abundance of loops at this location correlates with multiple CTCF bindings and CTCF motifs convergent to the stripe anchor. In other cells, the cohesin escaped from the arrest of the CTCF^[^ ^29^ ^]^ and established a near‐uniform distribution of loops until another CTCF, located ≈600 kb away from the stripe anchor, marked the end of the domain. These findings confirm that scGSLoop is capable of capturing the intrinsic heterogeneity in chromatin architecture due to looping dynamics, thereby providing deeper insights into the spatial organization of the genome and its functional implications.

To validate the accuracy of the predicted loops at the single‐cell level, we plotted the distribution of F1 score, precision, and recall for each set of loops predicted across different cells (Figure 5b). Affected by the cell‐to‐cell variability, loops in individual cells may differ significantly from the statistical ensemble represented by the reference loop list, making it difficult to achieve comparable accuracy with the consensus map. Nevertheless, the relatively high F1 score and precision indicates that the single‐cell loops captured by scGSLoop correspond with the overall conformation pattern of the genome as detected in bulk Hi‐C experiments, confirming the usability of scGSLoop in predicting loops at the single‐cell level.

### 2.6 Single‐Cell Loops Reveal the Key TF in Multi‐Connected Hubs

The detection of loops at the single‐cell level can provide more valuable insights into the complex mechanisms underlying chromatin conformation. To exemplify this, we employed single‐cell loops to identify 3D multi‐connected hubs^[^ ^28^ ^]^ in astrocytes (Tables S3–S6, Supporting Information), which allowed us to confirm the crucial role of a transcription factor (TF) in regulating hub structures.

**Figure 6:**
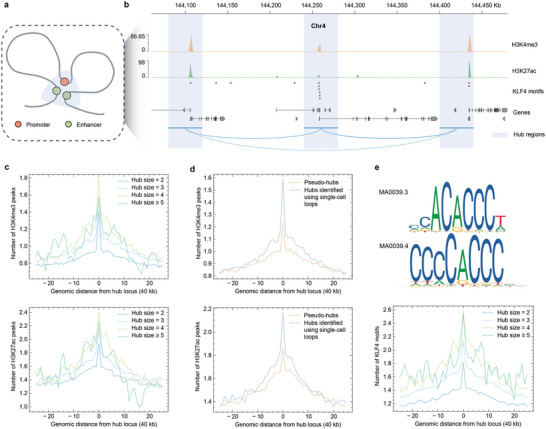
Characterization of 3D multi‐connected hubs in astrocytes using single‐cell loops detected by scGSLoop. a) Schematic diagram of multi‐connected hubs. b) An example genomic region that forms a multi‐connected hub of size 3. The red points represent KLF4 motifs at certain genomic coordinates. c) Histone modification profiles surrounding hub anchors of sizes 2, 3, 4, and above 5. A hub size of 2 represents a normal doublet loop. Upper part: H3K4me3; lower part: H3K27ac, same below. d) Comparison of histone modification profiles between the hubs identified using single‐cell loops and pseudo‐hubs generated from the consensus loop list. The pseudo‐hub group shown in this figure was obtained by subtracting the hubs identified using single‐cell loops from the set of all pseudo‐hubs. As such, they are two disjoint sets independent of each other. e) Analysis of KLF4 motifs near hub anchors of different sizes (ranging from 2 to 5 or beyond), with the upper part displaying position weight matrices (PWM) of KLF4 binding motifs retrieved from JASPAR^[^ ^35^ ^]^ and the lower part showing the average numbers of KLF4 motifs surrounding the hub anchors.

3D multi‐connected hubs are organizational units in the genome where multiple distal regulatory elements, such as promoters and enhancers, are brought proximal to interact with each other^[^ ^28^ ^]^ (**Figure** **6**a,b). Previous studies have corroborated that these structures are correlated with elevated transcription activity, indicating functional implications arising from collaborative communications among regulatory elements in multi‐connected hubs.^[^ ^36^, ^37^, ^38^ ^]^ ScHi‐C, as previously described in ref. [39], was leveraged as a standard for evaluating the putative multiway interactions predicted from bulk SPRITE^[^ ^40^ ^]^ and ChIA‐Drop,^[^ ^41^ ^]^ since the contacts in scHi‐C matrices are considered to occur simultaneously in the same nucleus.

Using scGSLoop, we were enabled to infer genome‐wide multi‐connected hubs by merging the single‐cell loops that share a common anchor harboring promoters. Specifically, a group of hub candidates was obtained from each cell, and the candidates from all cells were merged to create a comprehensive list. Finally, this list was intersected with the “pseudo‐hubs” identified using the consensus loops, resulting in a compilation of high‐confidence hubs (see Section 3; 40 kb resolution was adopted for the analyses in this section). As the single‐cell loops predicted by scGSLoop are clear of artifects and are more enriched with functional elements, the hubs inferred in this way were expected to exhibit consistent transcription activities in line with previous studies. We first examined the enrichment of histone modifications that act as markers for active promoters or enhancers (H3K4me3 and H3K27ac) around hub anchors (Figure 6c). The figure illustrates that both markers exhibited greater enrichment at hubs (hub size ⩾ 3) compared to doublet loop anchors (hub size = 2). In addition, we observed a general upward trend in the average number of peaks at hub anchors as the hub size increased, although this trend was disrupted when the hub size reached five. One possible explanation for this discrepancy could be the differences in the ratios of additive, synergistic, and redundant enhancers^[^ ^28^ ^]^ among these hubs.

To verify that the epigenetic features of the hubs were attributable to the single‐cell resolution, we compared the hubs identified using single‐cell loops against the pseudo‐hubs in terms of their histone modification profiles. Figure 6d illustrates the differences in H3K4me3 and H3K27ac enrichment between both sets. It is shown that the hubs derived from the single‐cell loops were more enriched with both modifications. The number of peaks at the anchors of the hubs identified using single‐cell loops was significantly higher compared to that of the pseudo‐hubs for both H3K4me3 and H3K27ac (Mann–Whitney *U* test *p*‐values 2.18 × 10^−38^ and 2.67 × 10^−17^, respectively). We also performed stratified comparisons with regard to hub size to verify that the observed increase of enrichment level was not due to specific hub sizes exhibiting exceptionally high enrichment, and the results confirmed that the histone modifications were more enriched in hubs identified using the single‐cell loops across different hub sizes (Figure S3, Supporting Information). These findings indicate that the ability of identifying 3D hubs indeed benefits from the model's capacity of detecting loops at the single‐cell level.

With hubs identified genome‐wide, we were enabled to examine the the overall enrichment of certain TFs and statistically investigate if a TF is associated with the formation and/or maintenance of multi‐connected hubs via significance tests. Previous studies have demonstrated that KLF4 plays an important role in the formation and regulation of enhancer hubs in mES cells.^[^ ^42^ ^]^ Here, we confirmed that KLF4 is also significantly enriched among multi‐connected hubs in human astrocytes. In particular, we utilized the human motifs for KLF4 binding from JASPAR^[^ ^35^ ^]^ and probed the distribution of the motifs surrounding hub anchors (Figure 6e). When the size of the hub increases, the average number of motifs at hub anchors displays a similar trend as that of the histone modification profiles (Figure 6c). Notably, the average number of motifs in multi‐connected hub anchors (hub size ⩾ 3) was significantly higher than that of normal doublet loops (Mann–Whitney *U* test *p*‐value =4.30 × 10^−15^). These findings potentially indicate that KLF4 may also perform critical functions in human astrocyte cells for mediating multi‐connected 3D hubs. These initial results, along with previous studies,^[^ ^42^ ^]^ suggest that KLF4 may be a promising target for future research aimed at understanding and treating diseases related to disrupted 3D hub formation.

## 3 Methods

### 3.1 Data Representation

The data representation in this study was inspired by the multi‐view nature of bulk Hi‐C data, as described in ref. [43]. Bulk Hi‐C data, which typically have large sequencing depths, can be represented as either images or graphs (i.e., the image view and the graph view). However, the Hi‐C image view collapses due to the extreme sparsity of single‐cell Hi‐C data. Therefore, when modeling scGSLoop, we used the graph view as the primary source of information. Additionally, we incorporated 1D sequence information, such as genomic distance measures and static DNA features, to improve the accuracy and efficiency of the model.

In the two‐stage pipeline of scGSLoop, we utilized Hi‐C data binned at different resolutions as input. For a single‐cell Hi‐C dataset *M* consisting of *N* cells, there are *N* × *C* *cis*‐contact maps, where *C* is the number of chromosomes of interest. For each chromosome *c* in the *l*th cell of the dataset, an undirected, unweighted chromosome graph $G^{\left(l,c\right)}$ is derived from the corresponding contact map *M* ^(*l*, *c*)^, where the adjacency matrix *A* ^(*l*, *c*)^ is represented using an element‐wise function 1 that outputs 1 for non‐zero inputs and 0 for zero inputs, as shown in the following equation

(1) $$A_{\mathrm{res}}^{\left(l,c\right)}=1\left(M_{\mathrm{res}}^{\left(l,c\right)}\right)$$

Here, the subscript res ∈ {LR, HR} denotes the bin size of the data, in which LR (low resolution) represents the contact maps of 100 kb bins and HR (high resolution) is the contact maps of 10 kb resolution. Prior to using these graphs in our framework, self‐loops are eliminated, which is equivalent to diag(A)=0.

In this study, graphs of LR were used for fast embedding generation (*K*‐nearest neighbor augmentation), whereas the HR graphs were fed to PC‐VGAE for loop edge prediction.

### 3.2 Two‐Step Pipeline for Loop Calling

ScGSLoop consists of two main components: an optional augmentation step and a loop prediction step. In the augmentation step, the low resolution (LR) chromosome graphs are used to generate a lower‐dimensional embedding for each cell. The augmented contact maps $M_{\mathrm{LR}}^{\left(l,c\right)}$ and $M_{\mathrm{HR}}^{\left(l,c\right)}$ are then constructed by averaging the Hi‐C contact maps for each cell and its *K* nearest neighbors in the embedding space. The augmented high resolution (HR) chromosome graphs are used in the loop prediction step, where a specialized graph autoencoder (PC‐VGAE) is employed to predict loop edges in each cell's chromatin structure.

The augmentation step is an optional component of the scGSLoop pipeline, designed to slightly increase the amount of information incorporated in contact maps and expand the number of candidate loops. However, if the sequencing depth is relatively high, this step may become slow and unnecessary, as the original information may be sufficient for scGSLoop inferencing. Therefore, we recommend skipping this step when the median number of contacts in individual cells exceeds 700 000. In this study, we predicted loops on the hPFC dataset using scGSLoop without the augmentation step. This allowed us to demonstrate the ability of scGSLoop to perform inference over un‐augmented chromosome graphs.

Together, these components enable the efficient and accurate prediction of chromatin looping across single cells, which are discussed in the next sections.

### 3.3 *K*‐Nearest Neighbor Augmentation

A self‐supervised variational graph autoencoder (VGAE) was adopted to project the nodes of the chromosome graphs into a lower dimensional space at the augmentation stage. A two‐layer GraphSAGE^[^ ^44^ ^]^ encoder compresses the 100 kb‐resolution genomic bins (i.e., the nodes of the chromosome graphs) into a multidimensional vector, and a generative decoder reconstructs the graph adjacency matrices from the latent vectors.

The VGAE for augmentation purpose only requires the 100 kb adjacency matrix itself as its supervision information. The training objective is to maximize the following objective:

(2) $$L=E_{q_{\phi }\left(Z|X,A\right)}\left[\log p_{\theta }\left(A|Z\right)\right]-\mathrm{KL}\left[q_{\phi }\left(Z|X,A\right)∥p\left(Z\right)\right]$$

where ϕ and θ represent the parameters of the encoder and the generative decoder, respectively. *X* represents the feature matrix of nodes. The posterior distribution *q*_ϕ_(*Z*∣*X*, *A*) is approximated by the encoder—For the *i*th node of the chromosome graph $G^{\left(l,c\right)}$, its latent variable **z**_*i*_ is sampled from the Gaussian distribution parameterized on $\mu_{i}$ and $\sigma_{i}$, which are both outputs of the GraphSAGE‐based encoder:

(3) $$q_{\phi }\left(z_{i}|X,A\right)=N\left(\mu_{i},\sigma_{i}^{2}\right)$$

Let **h**_*i*_ be the feature vector of node *i* and N(i) be the neighbor set of *i*, the forwarding rule of the GraphSAGE layers used in the encoder can be formulated as:

(4) $$h_{i}^{\prime }=W_{1}h_{i}+W_{2}\cdot {\mathrm{mean}}_{j\in N(i)}\left\{h_{j}\right\}$$

Reparameterization trick^[^ ^45^ ^]^ was adopted to sample from the posterior distribution. In Equation (2), KL[·∥·] denotes the Kullback–Leibler divergence, by optimizing which can the latent distribution get close to the presumed prior $p\left(z_{i}\right)=N\left(0,I\right)$.

The generative decoder performs variational inference to fit the posterior of the latent variables. We used a multilayer perceptron (MLP) as the decoder in this study:

(5) $$p\left(A_{ij}=1|z_{i},z_{j}\right)=\sigma \left(\mathrm{MLP}\left(\mathrm{concat}\left[z_{i},z_{j}\right]\right)\right)$$

in which σ denotes the sigmoid function.

The learned latent vectors of nodes were pooled to generate the mean vectors that represented the whole cells. A distance matrix was computed according to Euclidean metric in the latent space. A contact matrix was then augmented by averaging the non‐zero entries of the graph itself and its neighboring cells.

Although the augmentation step increased the density of interaction matrices, the augmented contact maps were still sparse, and therefore kept them suitable for graph‐based learning.^[^ ^43^ ^]^ On the other hand, the sparse representation was preserved so that the data could fit into the memory of a normal workstation, which was a vital factor of the lightweight workflow.

### 3.4 Proximity‐Aware Constrained Variational Graph Autoencoder

During the loop prediction stage of scGSLoop, a VGAE of comparable structure to the one employed in the augmentation phase was utilized to assign a probability score to each potential edge on the graph. This score was used to determine whether a candidate edge represented a chromatin loop. It is worth highlighting that, at this stage, the labels for training were reference loops obtained from bulk sequencing data (Section 3), not the adjacency matrices of chromosome graphs.

However, the application of VGAE on this scHi‐C loop calling task faces challenging problems during both the training process and the inferencing process. On the one hand, during the training stage of VGAE, a negative sampling technique is employed to create class‐balanced batches of data on each parameter update. The negative samples are randomly collected over all possible edges on the fully connected graph. However, in the context of Hi‐C loop calling, loops are most likely to appear in a much smaller range of genomic distance (e.g., <1 Mb). The hard examples (i.e., the edges that are difficult to classify) also tend to lie within this short genomic distance range. Using the vanilla negative sampling method, most negative examples are collected from the distances that are out of the target range and are easy for the model to distinguish, especially when locus positional information is provided as node features. Consequently, the model experiences information deficiency and produces a classification boundary that is too smooth, leading to an excessive number of false positive predictions.

On the other hand, the vanilla VGAE runs the risk of encountering memory overflow issues during the inference stage. While the sampling strategy allows VGAE to be trained sparsely, the model must produce a dense probability map that is the same size as the graph adjacency matrix. Unlike the augmentation step, the graphs used in the loop prediction stage contain ten times as many nodes, resulting in the probability map consuming 100 times more memory.

To tackle these issues, PC‐VGAE was developed as a solution. We incorporated two mechanisms into VGAE that are specifically geared toward scHi‐C loop calling, significantly enhancing the model's accuracy and scalability.

#### 3.4.1 Proximity‐Aware Negative Sampling

A proximity‐aware negative sampling mechanism was created to enhance the model's proficiency in classifying loop candidates within the desired genomic distances. Rather than stochastically sampling from all entries on the whole adjacency matrix, negative examples were sampled from non‐looping contacts (i.e., the contacts in the dataset that are not in the reference loop list) and other non‐contact entries within the target genomic distance. This approach enabled the information used for parameter optimization to better represent the local structural context, resulting in a more refined decision boundary for the neural network.

#### 3.4.2 Constrained Edge Prediction

We developed the constrained edge prediction method with the aim of avoiding memory overflow during inference. This approach restricts loop prediction to a limited number of discrete candidates, rather than creating a dense probability map for the entire adjacency matrix. Through the use of the constrained edge prediction mechanism, we assumed that the likelihood of chromatin loops occurring at non‐contact entries was zero. Consequently, the set of loop candidates was narrowed down to the contacts that were present in the raw or augmented Hi‐C interaction matrices. This mechanism converted the dense operation to a sparse operation, reducing the computational complexity and making the algorithm more efficient.

### 3.5 Local Degree Profile Features

The chromosome graphs G themselves are non‐attributed graphs where the nodes do not possess features. To accelerate convergence of the model, we augmented each LR chromosome graph using its local degree profile (LDP).^[^ ^46^ ^]^ LDP calculates a 5D feature vector for each node by analyzing the degree statistics of the node itself and its neighboring nodes. The LDP feature of the *i*th node can be formulated as follows:

(6) $$\begin{array}{c} x_{i}=\left[\deg\left(i\right),\min\left(\mathrm{DN}\left(i\right)\right),\max\left(\mathrm{DN}\left(i\right)\right),\mathrm{mean}\left(\mathrm{DN}\left(i\right)\right),\mathrm{std}\left(\mathrm{DN}\left(i\right)\right)\right] \end{array}$$

where DN(i) denotes the degrees of the neighbors of node *i*.

In scGSLoop, LDP was used to generate node features for graph learning in the augmentation module so that the performance of the network could be uplifted.

### 3.6 Static DNA Features

In the loop calling stage, static DNA sequence features were gathered for each genomic bin and used as node features for chromosome graphs. These features encompassed 3‐mer and 4‐mer statistics, as well as the count and orientations of CTCF motifs at each locus. Collectively, all these attributes composed a 322‐dimensional feature vector.

We retrieved the coordinates of the motifs of human (hg19) and mouse (mm10) from ref. [47], which were identified through scanning the corresponding genome assemblies annotated with FIMO.^[^ ^48^ ^]^ The identification of these motifs was accomplished by utilizing the position weight matrix (PWM) MA0139.1 from JASPAR.^[^ ^35^ ^]^

While the static DNA features are not directly encoded in scHi‐C data, they can be easily and quickly obtained through genome‐wide scanning of genome assemblies. It is worth noting that this scanning process does not necessitate any other data type, making it user‐friendly. In addition, we have included the static DNA features of common genome assemblies (hg19, hg38; mm9, mm10) in the released code, which can be readily utilized.

### 3.7 Genome Locus Positional Encoding

To encode the position of a locus within the chromatin during the loop calling stage, we employed a positional encoding layer similar to the one used in the transformer model.^[^ ^25^ ^]^ This layer vectorizes the genomic coordinates of each locus and integrates them with other existing features. The position of each locus in the DNA sequence is encoded as a *d*‐dimensional vector. Let *L* be the length of the sequence and *k* be the index of a locus, the positional feature matrix of shape (*L*, *d*) is defined as follows

(7) $$\begin{array}{cc} P\left(k,2i\right) & =\sin\left(\frac{k}{n^{2i/d}}\right) \end{array}$$

(8) $$\begin{array}{cc} P\left(k,2i+1\right) & =\cos\left(\frac{k}{n^{2i/d}}\right) \end{array}$$

where $0\leq i<\frac{d}{2}$ maps each dimension to sine and cosine functions, and *n* is a scalar parameter that was set to 10 000 in the original paper. This encoding scheme preserved the order information of the sequence data, which is crucial for our task. Moreover, this layer is non‐parametric and does not require training, which also contributed to the computational efficiency of scGSLoop.

### 3.8 Consensus Loop Set of Multiple Cells

The direct output of scGSLoop is the loop annotations of individual cells using a threshold of 0.5. To obtain the consensus loop set of each cell type, we aggregated the predictions on individual cell level by taking the sum of loop probabilities, and re‐scaled the summed probability values to the range of [0, 1]. Finally, a percentile threshold was adopted to filter the list to the final loop annotations. We also used the same blacklist of genomic regions to eliminate any invalid loops present in the consensus loop set.

### 3.9 Discovery of 3D Multi‐Connected Hubs

To identify hubs from single‐cell loops, we utilized a promoter‐centric algorithm. First, we coarsened the loops to a resolution of 40 kb and then searched for loop anchors containing promoters of known genes across the entire genome. We designated an anchor and its connecting anchors as a candidate hub if the promoter‐anchor was connected to two or more other loci. We followed the same procedure for the consensus loop set to identify pseudo‐hubs at the bulk level. To generate the final list of 3D multi‐connected hubs, we took the intersection between the single‐cell candidate hubs and the pseudo‐hubs.

It is worth noting that the pseudo‐hubs were derived from the high‐confidence loops that can be confirmed in the consensus. These loops were considered to be reliable and accurate representations of the 3D chromatin structures that were stably existing across multiple cells. Therefore, we considered the pseudo‐hubs as a standard for filtering the candidate hubs discovered from the single‐cell loops so that the infrequent gene regulation behaviors in single cells can be excluded.

### 3.10 Loop Reference List Called from Bulk Sequencing Data

We utilized the reference loop list provided in the SnapHiC paper^[^ ^21^ ^]^ to train and assess our models. In the case of the mES dataset, the reference list was composed of loops identified by HiCCUPS^[^ ^2^ ^]^ on in situ Hi‐C data, as well as significant interactions detected on bulk H3K4me3 PLAC‐seq, cohesin HiChIP, and H3K27ac HiChIP data using MAPS. For oligodendrocytes, microglia, and neuron cells in the hPFC dataset, the reference loops were called from H3K4me3 PLAC‐seq data using MAPS.

Furthermore, we employed the blacklist regions of hg19 and mm10 outlined in ref. [21] to eliminate any invalid loops from our final output.

### 3.11 Metrics for Performance Evaluation

To ensure a fair comparison between our method and SnapHiC, we utilized the same evaluation metrics as those described in ref. [21], which include precision, recall, and their harmonic mean *F*1 score. Precision is calculated by dividing true positives by the sum of false positives and true positives. It is expressed as TP / (TP + FP). Recall is the ratio of true positives to the sum of false negatives and true positives, expressed as TP / (TP + FN). *F*1 score is calculated using precision and recall, which can be fomulated as

(9) $$F1=2\times \frac{\mathrm{precision}\times \mathrm{recall}}{\mathrm{precision}+\mathrm{recall}}$$

We followed the approaches described in SnapHiC and allowed a 20 kb gap upstream and downstream of both sides of a bin pair. When calculating precision, predicted loops with both loop anchors fell into ±20 kb ranges of labels were counted as TP, while for the calculation of recall, the TP was the number of label loops falling into the predicted loops with ±20 kb slackness.

### 3.12 APA

Aggregate peak analysis (APA) was performed using FAN‐C.^[^ ^49^ ^]^ To carry out APA, we first aggregated individual cells to construct pseudo‐bulk contact maps. The aggregate command of FAN‐C was applied to the KR normalized maps, utilizing the ‐e option.

The *z*‐score was determined as per the methodology outlined in ref. [2]. Specifically, the mean and standard deviation were derived from the 6 × 6 submatrix located at the lower left corner of the aggregated loop matrix. A *z*‐score higher than 1.64 indicates the significant enrichment of chromatin loops.

### 3.13 Enrichment Analysis of ChIP‐Seq Peaks and TF Motifs

We conducted enrichment analyses on histone modification peaks (H3K4me3 and H3K27ac) as well as KLF4 motifs by quantifying the number of peaks or motifs present on loop/hub anchors. To do this, we extracted the anchors associated with the loops/hubs of a given size category (*k* = 2, 3, 4, etc.) and eliminated any duplicates. We then determined the enrichment by computing the mean number of peaks/motifs over these anchors. To plot the enrichment profile, as illustrated in Figures 4b and 6c–e, we calculated the enrichment scores for the genomic bins surrounding the anchors on the 1D DNA sequence in the same manner. In a profile figure, the *y*‐axis value that corresponds to *x* = 10 indicates the average number of peaks/motifs for the tenth genomic locus situated downstream of each anchor with size‐*k* hubs.

Following this approach, we were able to analyze the enrichment of histone peaks and KLF4 motifs without being influenced by the size of the hubs.

### 3.14 Implementation and Hardware Specifications

We used Cooler^[^ ^50^ ^]^ to store and read scHi‐C data. When using scGSLoop for a custom scHi‐C dataset, scGSLoop accepts .scool format as input, and outputs a .bedpe file for each cell. The neural networks were implemented using PyTorch and PyTorch Geometric.^[^ ^51^ ^]^ The hardware specifications of the system used in this study included an Intel(R) Core(TM) i7‐10700 CPU, 64 GB of memory, a hard disk drive (HDD), and a 10 GB NVIDIA GeForce RTX 3080 graphics card, all operating on the linux subsystem (WSL) of a Windows workstation.

### 3.15 Statistical Analysis

In this study, we employed SciPy^[^ ^52^ ^]^ to conduct hypothesis tests. Specifically, we used the two‐sided Wald test to examine the linear relationship between the number of cells and computational overhead (running time or memory consumption). Additionally, we employed the two‐sided Mann–Whitney *U* test to test for significantly higher enrichment in Figure 6d,e.

It is worth noting that no transformations were applied to the samples before conducting the significance tests. For both the Wald Test and the Mann–Whitney *U* Test, we set the threshold of significance at *p* < 0.05.

## 4 Discussion

In this study, we proposed a new paradigm of graph‐based learning on single‐cell Hi‐C data. Utilizing the proximity‐aware negative sampling, the GNN is capable of extracting more informative feature representations from local genomic contexts. Together with the constrained edge prediction mechanism, scGSLoop is able to detect loops accurately in a sparse fashion, facilitating fast and memory‐safe training and inferencing. Notably, our approach enables the annotation of loops at the single‐cell level. To the best of our knowledge, this is the first computational tool that can capture the cell‐to‐cell variability of chromatin loops. With such a tool, we are enabled to better sketch the landscape of 3D genome through the single‐cell lens.

As an application that leverages the multi‐view nature of Hi‐C contact matrices,^[^ ^43^ ^]^ scGSLoop adopted the graph view as the main data representation to operate on, while used the sequence view as an auxiliary source of information. Although the model is not sequence‐based, the information provided by the sequence view is crucial. The PC‐VGAE was designed based on the 1D distance on the sequence, and the node features of the graph were also derived from the DNA sequences of the genomic loci. The exceptional performance of scGSLoop further emphasizes the significance of incorporating multi‐view features in machine learning applications for Hi‐C data.

In a broader sense, incorporating the sequence view of Hi‐C data could offer a wealth of information beyond the simple static DNA features employed in this study. For example, Feng et al.^[^ ^53^ ^]^ integrated 1D epigenetic features to create a model that could impute low‐resolution bulk Hi‐C contact maps. However, this approach requires multiple epigenetic signal tracks (such as CTCF ChIP‐Seq, H3K27ac ChIP‐Seq, and ATAC seq) for each cell type or cell line as input. Consequently, using such information in scHi‐C loop detection could be prohibitively expensive, as the user would need to acquire these epigenetic features for each distinct cell type in the scHi‐C dataset, severely limiting the model's practicality. To ensure the ease of use of scGSLoop, we opted not to include this information in our model.

Apart from that, static DNA features can also be significantly improved by high‐capacity computational models. A recent breakthrough in gene expression prediction from DNA sequence involves the use of the transformer model, as seen in Enformer.^[^ ^54^ ^]^ This model extracts features and makes predictions based on the DNA sequence. Another example is the computational modeling of the genome's 3D structure directly from DNA sequences. Orca^[^ ^55^ ^]^ utilizes large‐scale convolutional neural networks (CNNs) to obtain high‐level sequence features that can be utilized to reconstruct the genome's 3D organization. Nevertheless, we chose not to adopt these methods due to the significant computational overhead they require to achieve outstanding performance, which could seriously impair the efficiency of scGSLoop.

Although we did not use these additional sequence features in this study due to usability concerns, they are highly likely to incorporate finer information that is useful for identifying chromatin loops at the single‐cell level. One potential future improvement of scGSLoop is to include epigenetic features, which could improve its ability to predict loops with greater functional significance. On the other hand, if greater computational resources are available, it may be possible to develop models that integrate pre‐trained parameters of Enformer or Orca to enhance the performance of scGSLoop.

## Conflict of Interest

The authors declare no conflict of interest.

## Supporting information

Supporting Information

Supporting Information

## Data Availability

In this study, we utilized scHi‐C data from mES cells, which were originally generated in ref. [9] and are publicly available at the Gene Expression Omnibus (GEO) under accession number GSE94489. Rather than processing the raw data ourselves, we utilized the pre‐processed contact matrices provided by the Tanay lab, which are available at https://github.com/tanaylab/schic2. For the hPFC dataset,^[^ ^14^ ^]^ raw data can be accessed at GEO under accession number GSE130711, while the processed Cooler files are available at https://salkinstitute.app.box.com/s/fp63a4j36m5k255dhje3zcj5kfuzkyj1/folder/82403061106. The ChIP‐Seq data for mES and astrocyte cells were acquired from ENCODE,^[^ ^56^ ^]^ with the following accession codes: mESC CTCF (ENCFF533APC), mESC H3K27ac (ENCFF274UIB), and mESC H3K4me3 (ENCFF974BMC); Astrocyte CTCF (ENCFF415WKV), astrocyte H3K27ac (ENCFF794VMY), and astrocyte H3K4me3 (ENCFF552LZP). The ChromHMM segmentations for mESCs^[^ ^57^ ^]^ and astrocyte^[^ ^58^ ^]^ cells are available at https://github.com/guifengwei/ChromHMM_mESC_mm10 and https://egg2.wustl.edu/roadmap/web_portal/chr_state_learning.html, respectively. Genome‐wide CTCF motif annotations of mm10 and hg19 were obtained from.^[^ ^47^ ^]^ The genome‐wide annotations of KLF4 motifs on hg38 were retrieved from JASPAR^[^ ^35^ ^]^ with motif IDs MA0039.3 and MA0039.4 and were converted to hg19 using liftOver. The chromatin loop annotations generated by scGSLoop at single‐cell and consensus levels are publicly available at Zenodo with the following doi: 10.5281/zenodo.7944922. The code of scGSLoop is open‐source and publicly available on GitHub at https://github.com/fzbio/scGSLoop.
