# Supplementary material for: A Lightweight Framework For Chromatin Loop Detection at the Single‐Cell Level
Source: Adv Sci (Weinh). 2023 Oct 10;10(33):2303502. doi: 10.1002/advs.202303502 (PMC10667817; doi:10.1002/advs.202303502)
Supplement: Supplementary file 1 — Supporting Information [file ADVS-10-2303502-s002.pdf]

ADVANCED SCIENCE
Open Access

Supporting Information

for *Adv. Sci.*, DOI 10.1002/advs.202303502

A Lightweight Framework For Chromatin Loop Detection at the Single-Cell Level

Fuzhou Wang, Hamid Alinejad-Rokny, Jiecong Lin, Tingxiao Gao, Xingjian Chen, Zetian Zheng, Lingkuan Meng, Xiangtao Li and Ka-Chun Wong**

Supplementary Figures

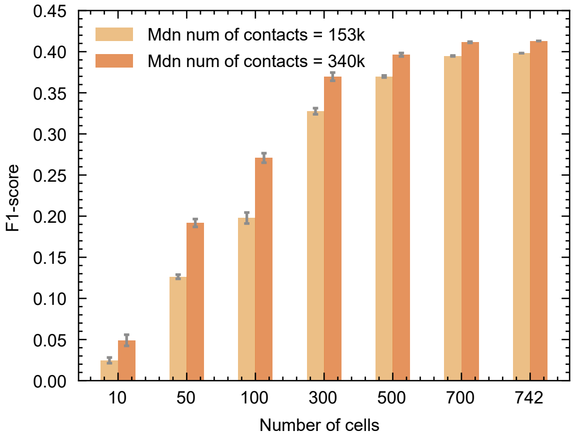

Figure S1. Evaluation of scGSLoop on the downsampled mES dataset, compared with the performance on the original mES dataset. A binomial distribution with $p = 0.45$ was applied to each entry on the contact map of the original mES dataset to make the sequencing depth of the downsampled dataset 10% as much as that of the training set (hPFC dataset).

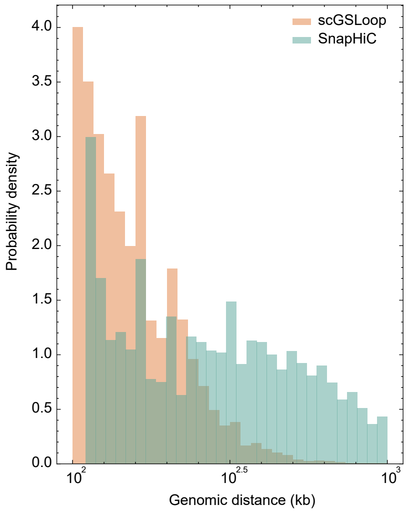

Figure S2. Loop size (i.e., the 1D genomic distance that a loop spans) distributions of the loops predicted by scGSLoop and SnapHiC.

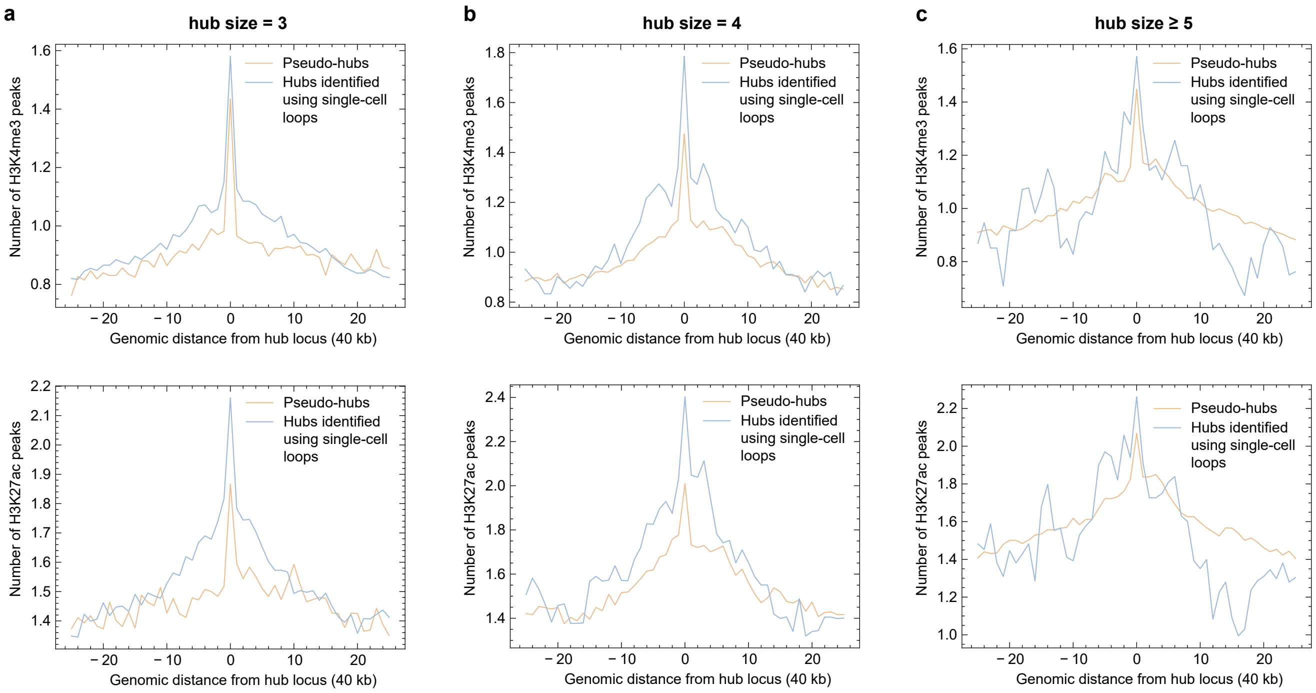

Figure S3. Stratified comparisons of histone modification profiles between the hubs identified using single-cell loops and pseudo-hubs across different hub sizes: **(a)** hub size = 3, **(b)** hub size = 4, **(c)** hub size $\geq$ 5.
